# Disproportionate infection, hospitalisation and death from COVID-19 in ethnic minority groups and Indigenous Peoples: an application of the Priority Public Health Conditions analytical framework

**DOI:** 10.1016/j.eclinm.2023.102360

**Published:** 2024-01-08

**Authors:** Patricia Irizar, Daniel Pan, Harry Taylor, Christopher A. Martin, Srinivasa Vittal Katikireddi, Niluka Wijekoon Kannangarage, Susana Gomez, Daniel La Parra Casado, Prashanth Nuggehalli Srinivas, Finn Diderichsen, Rebecca F. Baggaley, Laura B. Nellums, Theadora Swift Koller, Manish Pareek

**Affiliations:** a Faculty of Humanities, School of Social Sciences, University of Manchester, UK; b Department of Respiratory Sciences, University of Leicester, UK; c Leicester NIHR Biomedical Research Centre, UK; d Department of Infectious Diseases and HIV Medicine, University Hospitals of Leicester NHS Trust, UK; e Development Centre for Population Health, University of Leicester, UK; f Li Ka Shing Centre for Health Information and Discovery, Oxford Big Data Institute, University of Oxford, UK; g Department of Global Health and Social Medicine, King’s College London, UK; h MRC/CSO Social and Public Health Sciences Unit, University of Glasgow, UK; i World Health Organization, Geneva, Switzerland; j Department of Sociology, University of Alicante, Spain; k Institute of Public Health, Bengaluru, India; l University of Copenhagen, Copenhagen, Denmark; m Department of Population Health Sciences, University of Leicester, UK; n Lifespan and Population Sciences, School of Medicine, University of Nottingham, UK

**Keywords:** COVID-19, SARS-CoV-2, Ethnicity, Race, Indigenous, Health inequity

## Abstract

The COVID-19 pandemic has resulted in disproportionate consequences for ethnic minority groups and Indigenous Peoples. We present an application of the Priority Public Health Conditions (PPHC) framework from the World Health Organisation (WHO), to explicitly address COVID-19 and other respiratory viruses of pandemic potential. This application is supported by evidence that ethnic minority groups were more likely to be infected, implying differential exposure (PPHC level two), be more vulnerable to severe disease once infected (PPHC level three) and have poorer health outcomes following infection (PPHC level four). These inequities are driven by various interconnected dimensions of racism, that compounds with socioeconomic context and position (PPHC level one). We show that, for respiratory viruses, it is important to stratify levels of the PPHC framework by infection status and by societal, community, and individual factors to develop optimal interventions to reduce inequity from COVID-19 and future infectious diseases outbreaks.

## Introduction

People from ethnic minority and Indigenous groups and have been disproportionately affected by the COVID-19 pandemic.^1^, ^2^, ^3^, ^4^, ^5^, ^6^ The reasons for this are complex but relate to health inequity with large-scale social, political, and economic disadvantages that accumulate across the life course and are passed through generations.^7^^,^^8^

Ethnicity is a social construct. An “ethnic minority group” can meet one or more of the following criteria: the group is numerically smaller than the rest of the population; it is not in a [social, economic, or politically] dominant position; it has a culture, language, religion or ethnicity that is distinct from that of the majority; and its members have a will to preserve those characteristics.^9^ The ways in which people are categorised into ethnic groups are racialised social processes, which vary across socio-historical contexts, and despite being abstract in nature, these social processes can manufacture tangible harms for racialised groups. The authors acknowledge that dedicated United Nations human rights bodies have the mandate to promote and protect Indigenous Peoples’ rights, as well as differing national status due to treaty obligations in some countries.^10^

Ethnic inequities exist in relation to exposure and vulnerability to risk factors, access, experiences, and outcomes within healthcare institutions. They are rooted in experiences of racism, i.e., the process by which systems (structural racism), policies (institutional racism), actions and attitudes (interpersonal racism or racial discrimination) interact to structure opportunities that discriminate and disadvantage minoritised ethnic groups and Indigenous Peoples, whilst unfairly advantaging the majority group.^11^, ^12^, ^13^, ^14^, ^15^, ^16^, ^17^ These disadvantages against ethnic minority groups and Indigenous Peoples continue to persist, despite multiple local/national laws and legislations, or international human rights conventions and treaties that mandate the adoption of all necessary measures to eliminate all forms of racial discrimination.^18^

**Fig. 1:**
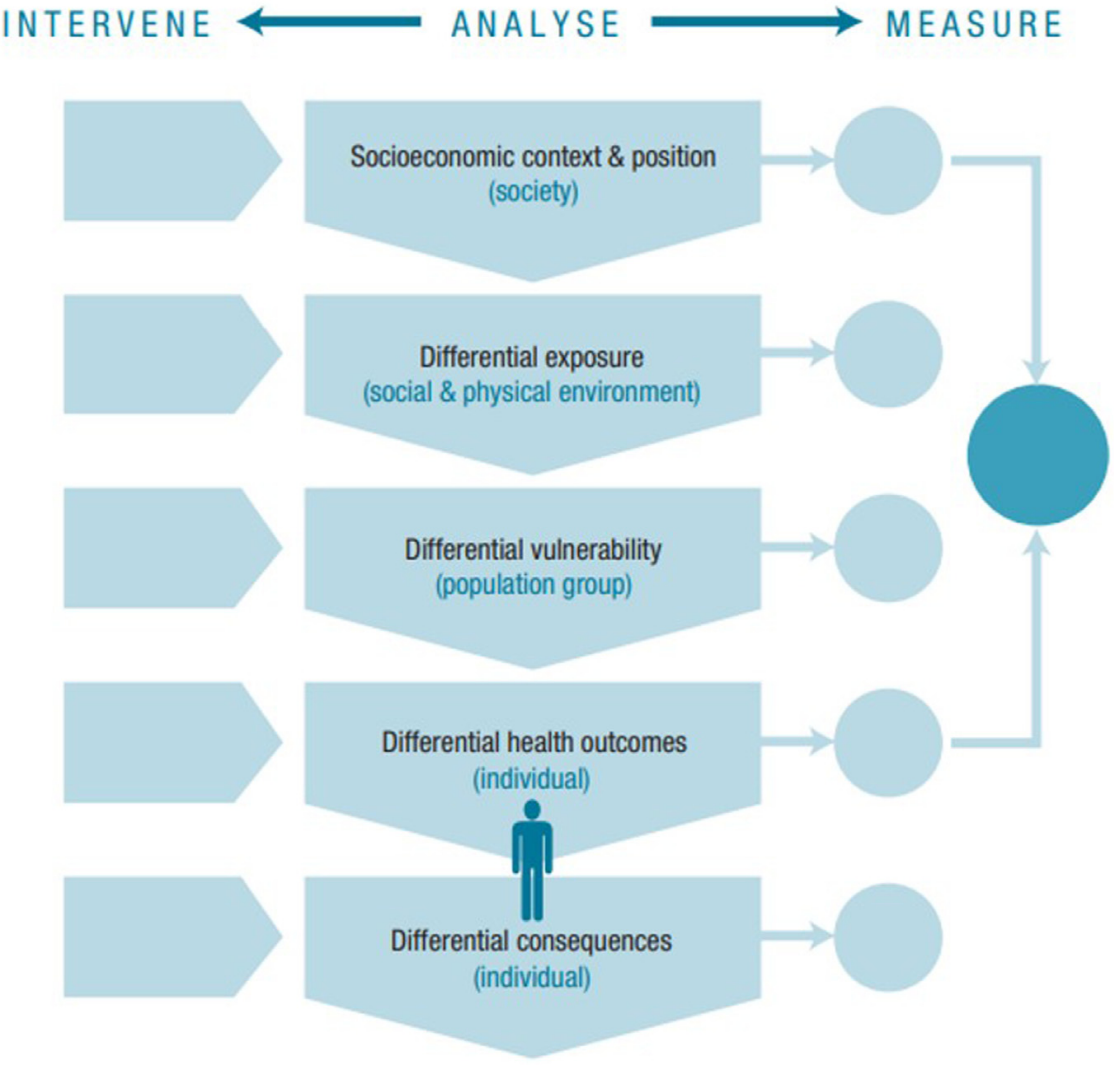
PPHC analytical framework, published in WHO (2010).

Several models of the processes that generate health inequities have been suggested for a variety of different health conditions.^19^ The Priority Public Health Conditions (PPHC) is a widely used five-level framework which can be applied practically to address the social determinants of health inequities (Fig. 1).^20^ The PPHC framework takes a holistic and value-driven view that socioeconomic position and conditions of daily life constitute the social determination of health and that they are crucial to explaining health inequities.^20^ Core dimensions of the PPHC were based on the original framework for understanding social origins of health inequities proposed by Diderichsen,^21^ and used by the Commission on Social Determinants of Health for its Priority Public Health Knowledge Network.^20^ Early on in the pandemic, expert academics presented frameworks drawing upon this original work, in the context of COVID-19 health equity for ethnic minority groups.^17^^,^^22^, ^23^, ^24^

In this manuscript, we reviewed the existing PPHC framework, examined evidence in relation to each level of the framework and using this information, extended the framework in a way that we believe could be further explored to address the health inequity issues relating to COVID-19 as well as for other respiratory viruses of pandemic potential. In July 2022, following a consultation with select experts in the field of health equity (see Acknowledgements), we decided that the PPHC framework was an equity framework with great potential for looking at COVID-19, because of its focus on socioeconomic context and position and differentials in exposure, vulnerability, access to services, outcomes, and social consequences. Our considerations have relevance globally in line with the Committee on the Elimination of Racial Discrimination (CERD) development of a General Recommendation related to racial discrimination and the right to health,^18^ and the WHO COVID-19 Strategic Preparedness and Response Plan Monitoring and Evaluation (SPRP M&E) framework,^25^ and were informed through discussions with experts across regions.

## Methods

### Search strategy and selection criteria

To examine the current evidence relating to COVID-19 and disproportionate outcomes within ethnic minority groups and Indigenous Peoples, we (PI and DP) conducted a non-systematic review of the literature. The authors have previously conducted three systematic reviews and meta-analyses of ethnic inequalities in COVID-19 health outcomes.^1^, ^2^, ^3^ The present manuscript syntheses the findings from these reviews and several additional reviews that were identified through searching key sources, including MEDLINE, PubMed, Scopus, and Google Scholar, using key words (e.g., “systematic review”, “meta-analysis”, “ethnic”, “racial”, “race”, “COVID-19”, “SARS-CoV-2”) to identify the key data syntheses regarding ethnic inequities in COVID-19 health outcomes up to November 2023, that were available in English (due to limited human and financial resources). We also searched reference lists and ‘cited by’ for each review that was identified. In addition, wider literature was searched to identify research relevant to the five levels of the PPHC, such as studies which investigated the reasons for ethnic inequities in COVID-19 health outcomes.

### Role of the funding source

The funders had no role in the study design; in the collection, analysis, or interpretation of the data; in the writing of the report; or in the decision to submit the paper for publication.

## Five levels of the PPHC analytical framework

The first level of the framework (Fig. 1), “socioeconomic context and position”, relates to the social relations between social positions (i.e., unequal social positions are the result of unequal social relations). Socioeconomic position is influential to health and can be defined by class, gender, ethnicity, Indigeneity, education, occupation, wealth, and income.^26^ For people from ethnic minority and Indigenous groups, socioeconomic context and position are affected by structural and institutional racism and racial discrimination, which are embedded within and between macro-level systems, institutions and ideologies to generate and reinforce inequities in material conditions as well as access to resources.^11^^,^^15^ In addition to the indirect pathways operating through socioeconomic position, discrimination also influences health through direct pathways.^16^ It is important to consider intersectionality when understanding ethnic inequities in health, as multiple aspects of social position (e.g., social class, gender, ethnicity) as well as spatial disadvantage (e.g., living in a rural and remote area with weak public service provision) can expose an individual to overlapping forms of discrimination and marginalisation that contribute to accumulated disadvantage.^27^

Structural and institutional racism and racial discrimination shape the landscape for the second level of the framework—“differential exposure”. Differential exposure is related to socioeconomic position, for example, financial insecurity, insecure employment, and lack of material resources may contribute to overcrowding and prevent the ability to self-isolate or the ability to main basic hygiene measures.^14^^,^^28^ In addition, minoritised ethnic groups are over-represented in many keyworker occupations who were on the frontline during the pandemic, increasing their risk of exposure.^29^ Previous evidence has also demonstrated ethnic inequities in exposure to other respiratory viruses, such as the H1N1 influenza pandemic.^30^

The third level of the framework is “differential vulnerability”, which, in the context of respiratory viruses, refers to whether those who are exposed become infected or not.^31^ Differential vulnerability is influenced by earlier infection, vaccination, stress, and other previous exposures during the life course. These factors are consequences of different forms of social deprivation, exploitation, marginalisation, exclusion, and segregation. For ethnic minority groups and Indigenous Peoples, racism and discrimination (including micro-aggressions) can manifest in heightened long-term stress responses, which can create epigenetic changes and increased allostatic load.^32^ This level of the framework also includes the treatment and consideration of ethnic minority groups within clinical research for vaccinations. Without proportionate inclusion of people from ethnic minority and Indigenous groups within clinical trials of SARS-CoV-2 vaccines, the evidence base supporting the effectiveness of such measures for these groups will remain poor.

The fourth level of the framework describes “differential health outcomes”. In this context, we are applying the PPHC framework to understand the course and medical consequences of SARS-CoV-2 in terms of severe disease and survival. In this level, we focus on whether those who are exposed to SARS-CoV-2 infection experience poor clinical outcomes (such as hospitalisation and death) or not. Experiences of racism and discrimination, as well as transgenerational accumulation of socioeconomic disadvantage, influence the biological and behavioural pathways to poor health.^12^^,^^14^^,^^33^ This increases the likelihood of multiple long-term conditions (comorbidities), which directly relates to poorer COVID-19 prognosis in those infected.^34^^,^^35^ The fourth level also reflects the quality of care received by certain groups, that affect their clinical outcomes. This includes inequitable access and/or suboptimal coverage of health services across the continuum of care (e.g., in access to testing, primary care, hospitals). Ethnic minority groups are often negatively impacted by poor quality or discriminatory treatment, inadequate interpretation services, and delayed or avoidance of help-seeking due to fears of undignified and disrespectful treatment.^13^ Similar to level three, this level of the framework covers the inclusion of ethnic minority groups and Indigenous Peoples in clinical research for COVID-19 treatment. Without appropriate inclusion in clinical research, there will be less trust within these groups to take medications, even if they are found to be beneficial.^36^

**Fig. 2:**
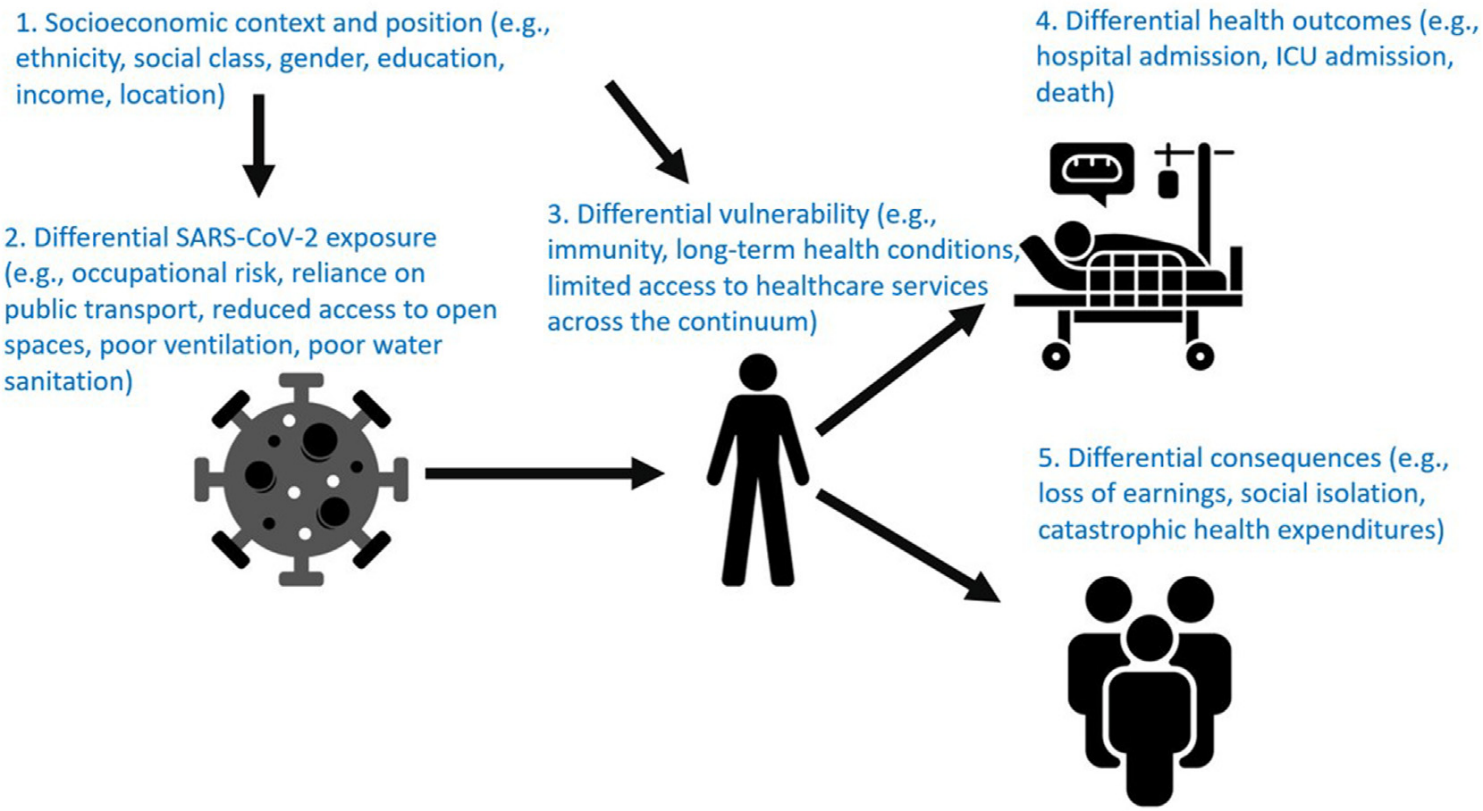
Potential endpoints following exposure to SARS-CoV-2 (black), and how the PPHC analytical framework (originally published by WHO in 2010) can be applied to study differential outcomes in ethnic minority groups (in blue). ICU, Intensive Care Unit.

The fifth level, “differential consequences”, reflects the unequal social and economic consequences (e.g., catastrophic health expenditures due to treatment costs,^37^ loss of earnings and loss of ability to work due to illness, social isolation and potential stigmatization) that infection, treatment and disability may have depending on labour markets and social policies. Disadvantaged groups disproportionally suffer from such consequences, particularly if adequate social protection measures are not in place. Fig. 2 summarises all the levels of the PPHC framework when applied to SARS-CoV-2 exposure and its consequences.

## Clinical outcomes of ethnic minority groups in relation to COVID-19: the evidence

### Early evidence from the first 6 months of the COVID-19 pandemic

An early rapid review published in May 2020 evaluated research and national surveillance reports on COVID-19, to identify studies of ethnicity data reporting patterns, associations, and outcomes. Only two of 29 publications reported ethnicity-disaggregated data at the time; both were case series without outcomes specific to ethnicity.^1^

A subsequent systematic review published in August 2020 found emerging evidence to suggest that ethnic minority groups were at an increased risk of SARS-CoV-2 infection, implying differential exposure (PPHC level two), severe COVID-19 (e.g., as indicated by admission to ICU), and mortality (PPHC level four).^2^ However, data published at the time were limited to the United States of America (USA) and the United Kingdom (UK). This review found that globally, although many countries were recording high numbers of confirmed COVID-19 cases and deaths, few were reporting these disaggregated by ethnic group.^2^

### Evidence up until December 2022

From 2020 to 2022, substantially more literature emerged that allowed for meta-analyses of clinical outcomes.^2^^,^^4^, ^5^, ^6^^,^^38^, ^39^, ^40^, ^41^ All meta-analyses found strong evidence of higher rates of infection within ethnic minority groups (PPHC level two), and some found a higher risk of COVID-19 hospitalisation in ethnic minority groups compared to majority groups (PPHC level four). However, many of these data syntheses focused on high-income countries only, presumably due to the lack of data from other settings, limiting their generalisability to global populations, and used broad terms relating to ethnicity (for example, Asian, but not South or East Asian) when performing their data synthesis.

### Evidence covering the whole pandemic period

The most recent meta-analysis was published in March 2023 and included 77 studies across the world, with 19 studies (i.e., 25%) obtained from low- and middle-income countries (LMIC), constituting over 200 million individuals in total.^3^ Given that the WHO declared an end to COVID-19 as a global health emergency shortly afterwards in May 2023, this work represents a summary of the most up-to-date literature regarding ethnic inequities, during the whole pandemic. Table 1 shows the number of eligible studies that were identified through the initial search (N = 579) and the regions where the studies were conducted, alongside the number of studies that were included in the synthesis (after removing studies that likely contained duplicate patient data). There remained very few studies which presented clinical outcome data disaggregated by ethnicity from Africa (four included studies) and Oceania (one included study) regions.

**Table 1:** Total number and percentage of studies including COVID-19-related outcomes disaggregated by ethnicity (into at least two different ethnic groups, e.g., ethnic majority group vs ethnic minority groups), stratified by region, before and after removing studies that likely include duplicate patient data.^3^

Region	Before de-duplication N (%)	Included in most recent meta-analysis^3^ N (%)
Africa	4 (1%)	4 (5%)
Asia	18 (3%)	13 (17%)
Europe	29 (5%)	19 (25%)
UK	112 (19%)	11 (14%)
North America	19 (3%)	7 (9%)
USA	369 (64%)	14 (18%)
South America	27 (5%)	8 (10%)
Oceania	1 (0%)	1 (1%)
**Total**	**579**	**77**

By now, there was substantial evidence of “differential exposure” (PPHC level two) as ethnic minority groups were more likely to test positive for infection and had higher seroprevalence (antibody tests indicating previous infection) compared to ethnic majority groups.^1^^,^^2^ Specifically within the meta-analysis, using terminology applied by the authors of the studies related to the specific ethnic groups in the countries where they did the research, compared to White people, Black (risk ratio [RR] = 1.8, 95% Confidence Intervals [CI] = 1.6–2.0), South Asian (RR = 3.0, 95% CI = 1.6–5.7), Mixed (RR = 1.6, 95% CI = 1.0–1.7), and Other (RR = 1.4, 95% CI = 1.0–1.8) ethnic groups were more likely to test positive for infection (estimates were concise with the exception of South Asian people, reflecting variation across studies and countries). In contrast to previous work, which only used molecular tests (PCR) to confirm infection status,^1^^,^^2^ the meta-analysis also found higher exposure for Hispanic people through seroprevalence studies only (RR = 1.9, 95% CI = 1.1–2.1), which may suggest differential access to testing early in the pandemic. Nevertheless, among population-based studies, there were also emerging evidence of marked inequities for most ethnic minority groups and Indigenous Peoples in severe disease (hospitalisation, ICU admission, and death), but these differences attenuated (or showed reduced risks for certain ethnic groups) when assessing prognosis (i.e., PPHC level four) among people infected with SARS-CoV-2.

## Applying the PPHC analytical framework in line with the evidence

Findings from the meta-analyses suggest that one of the key drivers of the greater risk of severe outcomes from COVID-19 among ethnic minority groups is due to a greater proportion of people from these groups becoming infected (PPHC levels two and three).^3^ However, there is a complex interplay of factors, influencing inequities at all levels of the PPHC framework. Evidence also indicates an increased risk of developing severe disease once infected for certain ethnic minority groups (PPHC level four). It is important to recognise that in the context of COVID-19, factors contributing to differential exposure are different from factors contributing to other levels of the PPHC framework. There is overwhelming evidence now that SARS-CoV-2 is spread mainly via the airborne route.^42^ Therefore, all that is required for an increased risk of exposure is a higher probability of coming into contact with others that are infectious. During the pandemic, many people from ethnic minority groups were working in lower paid jobs, had insecure employment and/or adverse working conditions, and were more likely to be keyworkers, making it more difficult to work from home or self-isolate.^29^^,^^43^ Ethnic minority groups were also more likely to be subject to poorer quality and overcrowded housing (which in turn decreases ventilation) with additional effort required to access essential goods and services, bringing increased risk of exposure.^16^^,^^44^

The third PPHC level also includes participation of ethnic minority groups in clinical research. A systematic review and meta-analysis, published in December 2022, of 122 COVID-19 clinical trials totalling 176,654 participants, found that Asian and Black participants were underrepresented in prevention trials, and Hispanic or Latino participants were overrepresented in COVID-19 treatment trials.^45^ A report by the UK National Institute for Health and Social Care Research found that ethnic minority groups comprise 9.3% of UK-based participants in COVID-19 studies, and 5.7% in vaccines^46^ (but ethnic groups other than White British comprise 26% of the UK population and ethnic groups other than “White” comprise 18% of the UK population).^47^ Whilst these analyses included only trials from the USA and UK, such trials have influenced policy in other countries, and thus, of global relevance.

Regarding the risk of severe disease once infected (PPHC level four), among hospitalised patients, certain ethnic groups are more likely to have specific comorbidities, e.g., diabetes and hypertension,^48^ that worsen short-term prognosis from COVID-19.^49^ More severe disease in these ethnic groups, however, could also be a consequence of differential access to healthcare, for example due to language barriers,^50^ migrant status,^51^ and health insurance coverage,^52^ that may mean they present later on in acute infection, when their symptoms are more severe. Many treatments for COVID-19, such as antiviral medication, awake prone positioning and non-invasive respiratory strategies, are most effective if started early on in illness and thus would be less effective in those presenting later.^53^, ^54^, ^55^

Finally, ethnic minority groups and Indigenous Peoples also suffered disproportionately as a result of becoming ill, e.g., income loss, job loss, food insecurity during the pandemic,^29^ which coincides with level five of the PPHC framework (differential consequences). The COVID-19 pandemic increased the utilisation of healthcare services, and countries without universal healthcare, disadvantaged groups may experience catastrophic costs of treatment.^37^ In addition, in countries that implemented government-mandated lockdowns, certain ethnic minority groups were over-represented in sectors that were negatively affected by lockdowns,^29^ and large surges in unemployment significantly undermined health insurance coverage.^56^ Health insurance impacts not only level five of the PPHC, but also levels three and four, as health insurance increases the likelihood of accessing services faster, subsequently impacting treatment outcomes.^56^ Pandemic control measures also had differential effectiveness by ethnicity. For example, after the implementation of a government-mandated lockdown in the UK, ethnic minority groups were more likely to continue testing PCR positive for SARS-CoV-2 compared to White groups, where PCR positivity decreased.^43^ Within medical occupations, ethnic minority groups in the UK were more likely to report lack of access to personal protective equipment (PPE)^57^ (or lack of access to suitable PPE; as PPE has been designed historically for the prototypical White male)^58^ despite these groups being more likely to care for the highest number of COVID-19 patients per shift and become infected.^59^

Studies from the USA and UK have found that ethnic minority groups are more likely to have persistent symptoms following initial infection (known by many names, such as long COVID, or post-COVID syndrome).^60^^,^^61^ Currently, long COVID is an emerging syndrome and the literature is evolving. However, there may be important differences in how long COVID manifests in different ethnic groups. At the very least, research is needed to understand better the mechanisms for these differences in symptoms and access to care. This is particularly important given the known inequities in SARS-CoV-2 vaccine access and uptake^62^, ^63^, ^64^ among some ethnic minority groups. Inequities in vaccine access and uptake may relate to financial situation (even when vaccines are free, given potential loss of earnings), car use, the ability to travel to vaccine centres, job security, and educational attainment.^65^ Qualitative data from healthcare workers in the UK suggest that lack of trust in government and employers, safety concerns due to speed of vaccine development, lack of ethnic diversity in vaccine studies and confusing and conflicting information about vaccinations contributed to hesitancy.^66^

## Limitations of the current PPHC framework when designing interventions to address inequities relating to COVID-19

Although the most useful of frameworks from the current literature, there remains key limitations for the use of the PPHC framework when policy-makers aim to apply it for COVID-19, or other respiratory viruses of pandemic potential. Firstly, the framework does not directly explore any differential effects of interventions for a given condition in any subpopulations, including ethnic minority groups.^22^ Interventions so far aiming to reduce SARS-CoV-2 spread such as lockdown, mass testing, contact tracing and vaccination have adopted a ‘one size fits all’ approach, with limited consideration of the specific cultural, social and language barriers that exist for ethnic minority groups, and an absence of auditing which interventions work, and which do not work at local and regional levels.^67^ By not targeting control at those who are at highest risk of infection, there may be severe implications on transmission of an infection across a whole population.

**Fig. 3:**
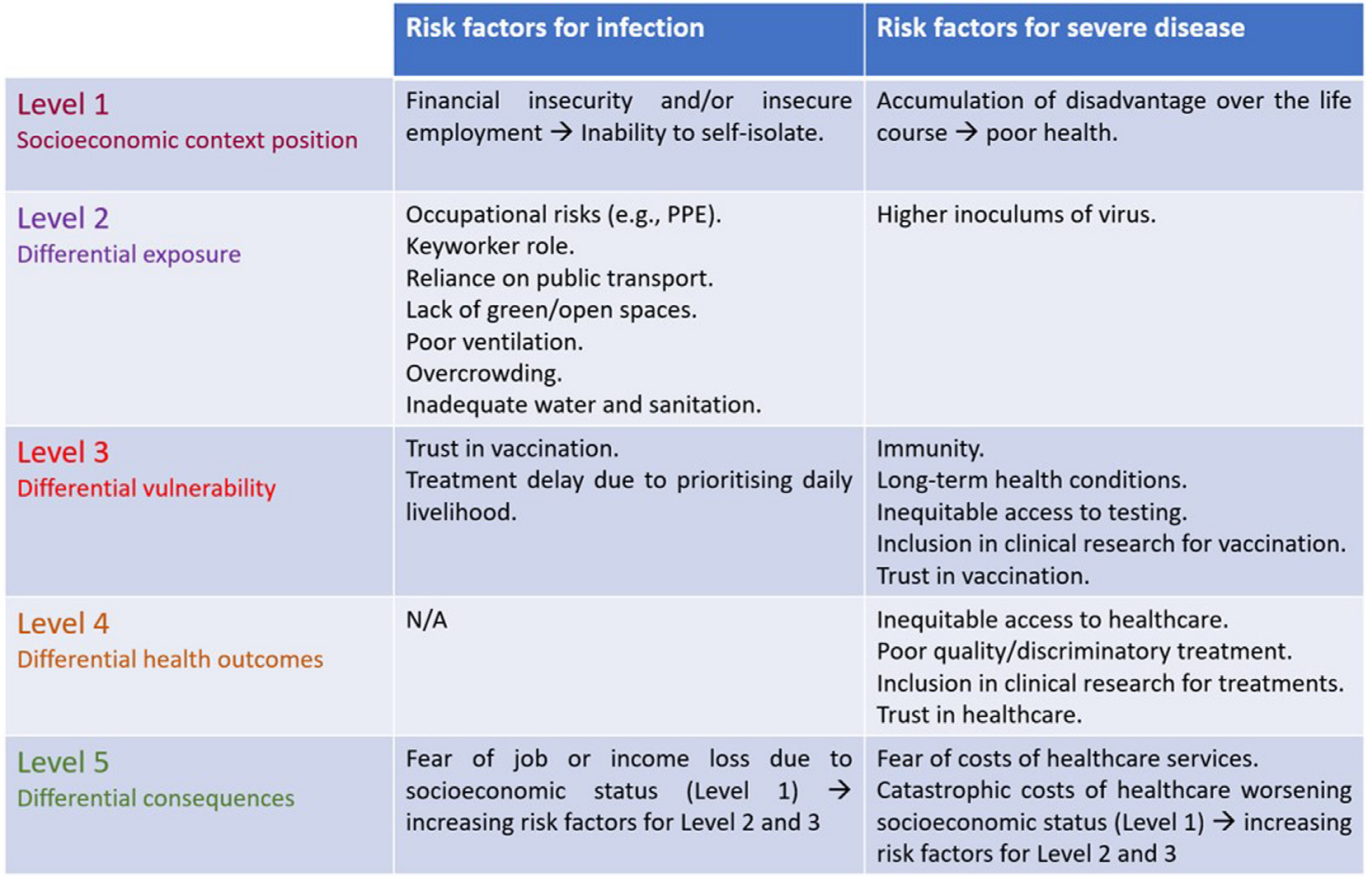
Summary of risk factors for infection and severe disease, stratified by the levels of the PPHC framework.

Secondly, the PPHC framework is limited in its ability to capture the inter-relationship of factors relating to social position (e.g., age, gender, socioeconomic position), and the intersection of pathways, as some individuals may have a greater risk of exposure only, whereas others may have multiple risks.^16^^,^^24^ Finally, the PPHC framework was originally applied to chronic noncommunicable diseases, such as cardiovascular disease, and communicable diseases such as Tuberculosis.^20^ For noncommunicable diseases, risk factors for their development are often the same as those which worsen prognosis. As mentioned above, risk factors for exposure to SARS-CoV-2 infection and other respiratory viruses with pandemic potential can be different from risk factors for the development of severe disease (Fig. 3). Risk factors for infection (e.g., socioeconomic position and occupational risks) require multifaceted interventions at societal levels to dismantle racist structures and systems which generate and reinforce ethnic inequities, whereas risk factors for severe disease (e.g., chronic health conditions, access to equitable healthcare, trust in vaccination and healthcare), are potentially more malleable through interventions at community and individual levels.

## Reframing the PPHC to measure the efficacy of interventions for COVID-19 and other respiratory viruses of pandemic potential

As we consider better global preparedness and response to pandemics, it is timely for innovative approaches using the PPHC framework to address the structural drivers of ethnic inequities in COVID-19 and other respiratory diseases caused by viruses of pandemic potential. We propose that each level of the framework may be driven by the interconnectedness of structural, institutional, and interpersonal racism, building on existing frameworks that were presented early in the pandemic.^17^^,^^22^^,^^23^ It is crucial that the PPHC should be applied in a way that recognises the presence of structural and institutional racism, as well as racial discrimination, and how they can co-occur and reinforce each other, deepening inequities across the life course, resulting in longstanding and persistent inequities in health.^23^ At the same time, in contrast to its application in chronic disease, the PPHC framework must be applied in a way that recognises that different kinds of interventions are required to address these inequities, before those from ethnic minority groups and Indigenous Peoples are infected, during the course of acute infection and in the months and years following initial infection.

**Fig. 4:**
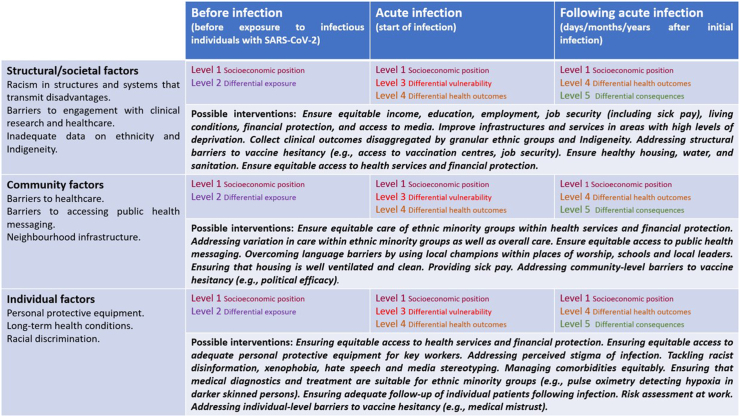
Figure showing how the levels of the PPHC framework apply for COVID-19 and other respiratory viruses of pandemic potential, stratified by infection status and factors relating to ethnic inequities at the structural/societal, community and individual level. Examples of interventions are also displayed to highlight the importance of intersectionality^27^ and consideration of ethnic inequity/socioeconomic position at every level, when designing interventions. Different colours highlight the different levels of the PPHC framework.

We have therefore extended the PPHC framework for pandemic preparedness, stratifying risk factors and relevant interventions across three phases (before infection, during infection, after infection) (Fig. 4). In recognition of the presence of ‘infection’ as a major trigger that results in downstream consequences for COVID-19, we combined the PPHC framework with the Haddon matrix, which is based on the socioecological model and epidemiologic concepts of disease causation that includes the presence of an external agent, a susceptible host and an environment that brings the agent and host together.^68^ We describe three phases where disproportionate outcomes can occur—before infection (exposure), during acute infection, and after infection, and show how various levels of the PPHC framework fit in these phases. Recognising the different levels of racism and how they intersect with each other, we present the three phases of infection against structural/societal, community and individual factors (Fig. 4). This new application provides clinicians, researchers, public health professionals and policymakers with a bespoke basis for identifying and analysing factors that influence differential outcomes of COVID-19 and other respiratory viruses of pandemic potential in ethnic minority groups and Indigenous Peoples, through an equity lens.

We consider level 1 of the PPHC framework (socioeconomic position, brown text in Fig. 4) to be prevalent across all phases of infection and across all societal, community, and individual factors. Interventions should therefore always consider addressing systemic inequity and structural racism which is intrinsically linked to this. Level 2 (differential exposure) is most relevant before infection (as demonstrated by the purple text within Fig. 4) and therefore should be considered in interventions early on during a pandemic (for example, addressing the barriers for adhering to lockdown). Level 3 (differential vulnerability, red text in Fig. 4) is most relevant during infection at all levels and needs to be considered within healthcare settings when people present to hospital, in addition to the community (for example, ensuring that ethnic minority groups and Indigenous Peoples do not present later in illness). Level 4 (differential health outcomes, gold text, Fig. 4) is most relevant both during acute infection and following infection; and Level 5 (differential consequences, green, Fig. 4) is most relevant following infection. Different interventions (examples given in Fig. 4) should intersect at societal, community, and individual levels, to address the multifaceted levels of discrimination and marginalisation. Some interventions (such as addressing vaccine hesitancy) will affect all phases and should be a focus to generate the most impact.

There are three key advantages of extending the PPHC framework for pandemic preparedness in this way. Firstly, there is a clear need to invest in gathering further evidence of what interventions are effective for eliminating inequities. Our framework allows for objective measurement and audit of interventions at different stages of an outbreak. Early on in an outbreak, before many have been infected, it would make sense to focus on reducing exposure. If an outbreak fails to be controlled, then it would be important to focus on interventions that can reduce differential vulnerability and health outcomes. Interventions which would reduce differential consequences come often in parallel, for example, fear of financial hardship as a consequence of diagnosis and treatment may influence susceptibility to inequities in health outcomes. Second, by recognising the importance of infection as a major trigger for the downstream consequences of a new outbreak, policy-makers should make all efforts to define infection transmission pathways early on during an outbreak. The main route of transmission will influence the interventions that are required to protect those from infection. Finally, the framework recognises how different interventions exist at a spectrum of structural, community and individual levels. At a structural level, interventions will require long-term funding and political investment that addresses the root of inequities, whilst individual level interventions are more short term.

Our extension of the PPHC framework can be applied globally, in line with the WHO COVID-19 Strategic Preparedness and Response Plan Monitoring and Evaluation (SPRP M&E) framework.^25^ The SPRP M&E framework calls for the transparent monitoring of COVID-19 response activities, through the production of systematic assessments and analyses of response activities (enabling information sharing), which can be compared against the epidemiological progression of the pandemic.^25^ This is paramount for recognising lessons that can be learned regarding health inequity for ethnic minority groups. As presented in Fig. 4, many of the risk factors are a direct reflection of policy decisions that have not invested in specific communities.

In recognition of the importance of addressing level 1 of the PPHC framework, we call for a better frameworks nationally and regionally for the collection of ethnicity data, that must involve ethnic minority groups in decision-making processes.^69^ These frameworks should prioritise collecting sufficient data with statistical power to enable stratification by ethnicity as well as other factors permitting an intersectional lens (backed by strong data protection measures), to ensure the identification of differential risks and disease pathways. Additionally, these frameworks should depend on the cultural and historical (including post-colonial) context for each country, and there is a clear need for data in regions where local ethnic identities are often not recognised as ‘ethnic’ groups (e.g., Indigeneity and caste identities in India). Data on local ethnic identities are typically not sufficient to enable disaggregated analyses and synthesising global data in a valid way is a trade-off for understanding its granularity within specific contexts. It is important to highlight that most of the presented research is from the US and UK (and is limited to published literature available in English), which, along with the primarily UK authorship of this paper, limits the understanding of ethnic inequities in different global contexts; a wider ethnic and global authorship would enrich this further. We must move away from the preference for biomedical data, which create bias in knowledge by not valuing the indigenous production of evidence and data sources that may not match Western academic criteria, otherwise we risk moving forward in a state of ignorance.^70^

## Outstanding questions

We present an extension of the PPHC, in line with socio-ecological models, to understand the intersecting pathways in the context of COVID-19 health equity for ethnic minority groups. There is clear evidence of differential exposure for ethnic minority groups, largely driven by socioeconomic context and position, which also influence differential vulnerability and differential consequences. Fundamentally, these pathways are a consequence of deeply entrenched racism and racial discrimination that lead to longstanding health inequities among ethnic minority groups, that persist across the life course and are transmitted across generations. We show that it is important, in the context of COVID-19 and other respiratory viruses of pandemic potential, to stratify the PPHC framework by both infection status (before, during acute infection and following infection) and by societal, community, and individual factors in order to develop optimal interventions to reduce inequity. In line with the SPRP M&E framework, the PPHC when presented in this way can be applied to identify intervention points, that can help the prioritisation of response activities and inform decision-making as we come out of the pandemic.^25^ There are clear opportunities for interventions focused on explicitly addressing racism and racial discrimination. In addition, interventions that support the delivery of universal services and in ways that address compounding and intersecting drivers of exclusion and marginalisation are required.^71^

## Contributors

PI and DP contributed equally to the conceptualisation, visualisation, writing of the original draft, and revising of the final manuscript. DK, HT, CM, FD, DLPC, PNS, and SVK made substantial contributions to critical review, editing, and revision of the manuscript. NWK, SG, RB, and LN contributed substantially to the conceptualisation, critical review, editing, and revision of the manuscript. TSK and MP contributed equally to supervision, conceptualisation, critical review, editing, and revision of the manuscript. TSK identified the need for this paper, building on previous work with the PPHC framework on inequities in COVID-19, and oversaw the paper’s commissioning. All authors have read and approved the final version of the manuscript. All authors had final responsibility for the decision to submit for publication.

## Declaration of interests

SVK was co-chair of the Scottish Government’s Expert Reference Group on Ethnicity and COVID-19 and a member of the UK Scientific Advisory Group on Emergencies (SAGE) subgroup on ethnicity. All other authors report no conflicts of interest.
